# Telomere length and human hippocampal neurogenesis

**DOI:** 10.1038/s41386-020-00863-w

**Published:** 2020-09-13

**Authors:** Alish B. Palmos, Rodrigo R. R. Duarte, Demelza M. Smeeth, Erin C. Hedges, Douglas F. Nixon, Sandrine Thuret, Timothy R. Powell

**Affiliations:** 1 Social, Genetic and Developmental Psychiatry Centre, Institute of Psychiatry, Psychology and Neuroscience, King’s College London, London, UK; 2 Division of Infectious Diseases, Weill Cornell Medicine, Cornell University, New York, NY USA; 3 Basic and Clinical Neuroscience, Institute of Psychiatry, Psychology and Neuroscience, King’s College London, London, UK; 4 Department of Neurology, University Hospital Carl Gustav Carus, Technische Universität Dresden, Dresden, Germany

**Keywords:** Senescence, Adult neurogenesis, Genetics

## Abstract

Short telomere length is a risk factor for age-related disease, but it is also associated with reduced hippocampal volumes, age-related cognitive decline and psychiatric disorder risk. The current study explored whether telomere shortening might have an influence on cognitive function and psychiatric disorder pathophysiology, via its hypothesised effects on adult hippocampal neurogenesis. We modelled telomere shortening in human hippocampal progenitor cells in vitro using a serial passaging protocol that mimics the end-replication problem. Serially passaged progenitors demonstrated shorter telomeres (*P* ≤ 0.05), and reduced rates of cell proliferation (*P* ≤ 0.001), with no changes in the ability of cells to differentiate into neurons or glia. RNA-sequencing and gene-set enrichment analyses revealed an effect of cell ageing on gene networks related to neurogenesis, telomere maintenance, cell senescence and cytokine production. Downregulated transcripts in our model showed a significant overlap with genes regulating cognitive function (*P* ≤ 1 × 10^−5^), and risk for schizophrenia (*P* ≤ 1 × 10^−10^) and bipolar disorder (*P* ≤ 0.005). Collectively, our results suggest that telomere shortening could represent a mechanism that moderates the proliferative capacity of human hippocampal progenitors, which may subsequently impact on human cognitive function and psychiatric disorder pathophysiology.

## Introduction

Patients with schizophrenia, bipolar disorder and major depressive disorder, have an average life expectancy approximately a decade lower than the rest of the population [1–3]. This statistic primarily reflects the higher prevalence of comorbid age-related diseases, including coronary artery disease, diabetes and dementia, which contribute to early mortality [3, 4]. Epidemiological findings such as these, have prompted researchers to consider whether faster ageing represents a core component to the pathophysiology of psychiatric disorders, or whether it represents the consequences of unhealthy lifestyles and stressful experiences, more common amongst those diagnosed with a psychiatric disorder [5, 6].

Telomere length is one biological marker which has been used to study rates of cell ageing amongst psychiatric disorder patients [7]. Telomeres are DNA repeat structures found at the ends of chromosomes, which in humans, comprise of a six-nucleotide repeat sequence (TTAGGG) [8]. Telomeres are vital for maintaining chromosomal stability [9] and have been shown to regulate a cell’s ability to replicate via mitosis [10]. Telomere length gets shorter with each somatic cell division due to the inability of DNA polymerase to fully replicate the 3′ end of the new DNA strand during DNA replication; a phenomenon known as the *end-replication problem* [11]. Furthermore, telomerase activity [12], oxidative stress [13], genetics [14] and specific environmental factors, such as stress and exercise [15], moderate telomere length and the rate at which it shortens. When a telomere reaches a critically short length, the cell stops dividing and reaches a state of cellular senescence, often referred to as the ‘Hayflick limit’ [16]. The Hayflick limit has been demonstrated in a variety of adult cell types in vitro, including fibroblasts [10], endothelial cells [17] and lymphocytes [18], whereby cells exhibit progressively shorter telomeres over increasing passages (or ‘population doublings’), a gradual reduction in their ability to proliferate, a reduced propensity to undergo programmed cell death, and a maladaptive proinflammatory phenotype [19]. The reduced replicative capacity of ageing cells is hypothesised to contribute to age-related tissue-level pathology in vivo; as old, damaged cells, can no longer be replaced with new, healthy cells [20].

In the context of psychiatry, meta-analyses reveal that, in general, psychiatric disorder patients exhibit shorter leukocyte telomere lengths relative to unaffected individuals of equivalent ages, which could contribute to the increased burden of age-related disease [21–24]. In addition to shorter telomere lengths, psychiatric disorder patients frequently exhibit neurological differences such as smaller hippocampi [25–27], and some studies have suggested a relationship between shortened telomere length and psychiatric disorder neuropathology [28–31].

The hippocampus is a brain structure important in cognition and mood regulation that is capable of adult neurogenesis due to the retainment of neural progenitors in the dentate gyrus; a specialised niche that allows progenitors to form new, functional neurons [32]. Most cross-sectional studies [29, 33–35], though not all [36–38], have reported positive associations between peripheral telomere length and hippocampal volume, or between peripheral telomerase activity and hippocampal volume [39]. Recent longitudinal data also support this relationship, demonstrating that greater leukocyte telomere shortening in elderly individuals is associated with a steeper loss in hippocampal volume [40]. Intriguingly, there is also a positive relationship between telomere length and cognitive performance [29, 31], and it has been hypothesised that telomere length contributes to this association by moderating the rate of adult neurogenesis [41]. This hypothesis is supported by various lines of evidence. First, chronological age and mood dysfunction is associated with both shorter telomere length in hippocampal tissue, and reduced rates of hippocampal neurogenesis [42–46]. Consequently, telomere shortening in proliferating neural cell populations might be driving tissue-level differences in telomere length observed in the hippocampus. Second, reduced rates of hippocampal neurogenesis correlate with poorer performance in the Morris water maze task [47], and in visual pattern discrimination tasks [48], indicative of cognitive dysfunction; as well as reduced swim time in the forced-swim test, indicative of depression-like behaviour [49]. Third, reductions in the expression of genes that regulate telomere length in the adult hippocampus in conditional knockout mouse models recapitulate age-related impairments to hippocampal neurogenesis, cognitive performance and behaviour, suggesting that telomere function is a key regulator of age-related changes to neurogenesis and cognition [50, 51]. Despite these insights, to-date, there have been no studies investigating the impact of telomere shortening on *human hippocampal* progenitor cells, nor its downstream relationship to cognition and psychiatric disease. This is largely due to the inaccessibility of the dentate gyrus; the fact that there are no validated peripheral biomarkers or neuroimaging tools to assess rates of hippocampal neurogenesis in vivo, in association with cognition and psychiatric conditions; and methodological challenges, like reliably immuno-staining post-mortem brain samples in large numbers [52].

The current study employed a systematic, multidisciplinary approach which aimed to model the effects of telomere shortening on human hippocampal neurogenesis, and subsequently its relationship to cognition and psychiatric disorder risk. First, we modelled telomere shortening using a serial passaging protocol that recapitulates the end-replication problem, in a human hippocampal progenitor cell line. We confirmed that reductions in telomere length were associated with lower levels of cell proliferation, without affecting the ability of cells to differentiate into neurons or glia. Second, complementary RNA-sequencing data revealed 3281 transcripts which change in association with telomere shortening. Of these, the 1594 downregulated genes strongly overlap with genes implicated in cognitive function and schizophrenia, and to a lesser extent bipolar disorder. Our work suggests that in addition to the established role of telomere length in age-related pathology, it may also play a role in psychiatric disorder neuropathophysiology via its effects on hippocampal progenitor cell proliferation.

## Materials and methods

### Human hippocampal progenitor cell line

An existing multipotent human foetal hippocampal progenitor cell line, HPCOA07/03 (ReNeuron, UK), was used to model human hippocampal neurogenesis in vitro, as used previously by our team [53–56]. These cells proliferate in the presence of growth factors, and upon their removal, differentiate into PROX-1 positive neurons, and glia. For further details on the cell line and culture conditions for proliferating and differentiating cells, see Supplementary information, S1, S2.

### Modelling telomere shortening via the ‘end replication problem’

As in other in vitro systems [17, 18, 57], we modelled telomere shortening by serially passaging cells. Serial passaging allows cell populations to double numerous times, and facilitates telomere shortening resulting from the end-replication problem (the incomplete synthesis of chromosome ends during DNA replication).

**Fig. 1:**
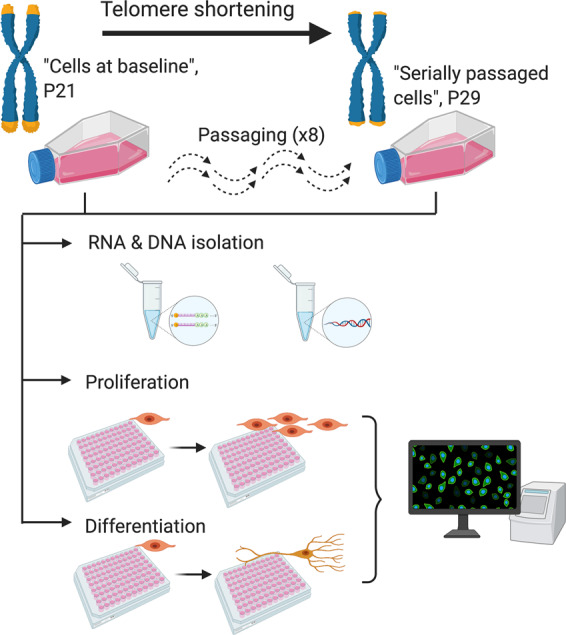
Protocol summary. A summary of our in vitro protocol, which considered telomere length, mRNA expression, cell markers of proliferation, and cell markers of differentiation, in association with cell passaging.

Our experimental protocol utilised four cryovials corresponding to four subcultures of cells (biological replicates), which were revived in separate T25 flasks and grown in proliferating medium. Once confluent, the cells were passaged onto a T75 flask under the same conditions, see Supplementary information, S1. ‘Cells at baseline’ in our model corresponded to those utilised at the start of our study, which had undergone fewer cell divisions and which have a lower passage number (P21). Subsets of cells were then, either isolated and pelleted for DNA and RNA extraction (telomere length assessment and RNA-sequencing); seeded onto two 96-well plates for proliferation and differentiation assays (immunocytochemistry); or seeded onto a new T75 flask for subsequent serial passaging, see Fig. 1. Passaging occurred once cells were 80–90% confluent (~48 h), as confirmed by cell count. At each passage, cells were reseeded at a density of 2 × 10^6^ cells/T75 flask to ensure an approximately consistent number of population doublings across biological replicates. ‘Serially passaged cells’ in our model corresponded to a subset of P21 cells that underwent eight subsequent passages (P29), where again we isolated DNA and RNA, or submitted cells to a final proliferation or differentiation assay, Fig. 1.

### Proliferation and differentiation assays (immunocytochemistry)

To compare differences in cell marker levels between cells at baseline and serially passaged cells, we performed immunocytochemistry using an established 3-day proliferation protocol performed on 96-well plates, where we assayed the proliferation markers BrdU and Ki67, and the apoptosis marker caspase-3 (CC3). We also assessed differentiation as part of a 7-day protocol, where we assayed neuronal markers, doublecortin (DCX) and microtubule-associated protein 2 (MAP-2), the astrocyte marker, S100β and apoptotic marker, CC3. See Supplementary information, S3–S5 for further details.

### Nucleic acid extraction

Approximately 50% of the cell suspension obtained during passaging was pelleted and stored at −80 °C at the beginning (passage 21; cells at baseline) and end of experiments (passage 29; serially passaged cells). DNA and RNA were extracted using the Qiagen AllPrep DNA/RNA/Protein Mini Kit (Qiagen, Hilden, Germany), see Supplementary information, S6, for further details.

### Telomere length measurements

Relative telomere length was quantified using DNA samples and a modified version of the quantitative Polymerase Chain Reaction protocol described by Cawthon [58], as used by our lab previously [5, 7, 14, 29], see Supplementary information, S7, for further details.

### RNA-sequencing

Library preparation and RNA-sequencing was performed at The Genomic Centre, King’s College London. Briefly, total RNA samples were submitted to a DNAse treatment using the DNA-free™ DNA Removal Kit (Invitrogen, California, USA). Subsequently, 300 ng of total RNA from each sample was submitted for ribosomal RNA depletion using the NEBNext rRNA Depletion kit (New England Biolabs, Massachusetts, USA), and RNA-Seq libraries were constructed using the NEBNext Ultra II Directional RNA Library Prep Kit for Illumina. The samples were sequenced in a HiSeq 4000 sequencing system (Illumina). Raw reads were downloaded and processed using a systematic approach, see Supplementary information, S8. Differential expression analysis was performed to compare cells at baseline versus serially passaged cells (*N* = 4 biological replicates per condition) using the Wald test in DESeq2, controlling for biological replicates. Log2 fold-changes were shrunk using apeglm [59], and the false discovery rate (FDR) correction was used to control for multiple comparisons. Gene expression differences were considered significant if *P*_FDR_ < 0.05.

### Gene ontology (GO) enrichment

To understand which biological mechanisms were being affected in our cell model in association with telomere shortening, we separately entered genes (*P*_FDR_ < 0.05) showing an increase in expression, and those showing a decrease in expression, into FUMA [60]. The GENE2FUNC tool performs a gene-set enrichment analysis, where gene sets were defined by The Molecular Signatures Database. We included all transcripts surviving DESeq2’s internal filtering criteria as our background list (i.e. all genes expressed in the samples). Multiple testing correction was performed using the FDR method, and GO terms were considered significant if P_FDR_ < 0.05.

### Gene-set enrichment analysis

We tested for a genetic overlap between upregulated and downregulated genes affected in our cell ageing model and those implicated in major depressive disorder [61], bipolar disorder [62], schizophrenia [63] and general cognitive function [64], using MAGMA [65] and publicly available GWAS summary statistics, see Supplementary Information, S9.

### Foetal brain samples

We performed a complementary experiment using foetal brain samples in order to clarify whether telomere shortening occurs in the human brain during early development in accordance with gestational age, and therefore, whether our cell model could also be recapitulating a neurodevelopmental cell process. Brain tissue was obtained frozen and had not been dissected into regions. Half of the brain tissue from each individual foetus was homogenised for subsequent genomic DNA extraction, which was performed by standard phenol-chloroform procedures as described previously [66]. Sample sex was determined via PCR amplification [66]. In total, DNA was available from 37 females and 48 males (*n* = 85). Gestational ages were estimated using foot and knee to heel length measurements. Gestational ages ranged from 75–161 days, with a mean gestational age of 107.96 (S.D. = 16.12). Telomere length was quantified as above, and as described in Supplementary Information S7. The human embryonic and foetal material was provided by the Joint MRC/Wellcome Human Developmental Biology Resource (www.hdbr.org). Ethical approval for the HDBR was granted by the Royal Free Hospital research ethics committee under reference 18/NE/0290.

### Statistical analyses

Differences in telomere length between the baseline condition and serially passaged cells was determined using a two-tailed independent-samples *t*-test. Similarly, differences in the expression of cell markers were determined using two-tailed independent-samples *t*-tests, followed by a Bonferroni correction to account for the total number of markers assayed in either the proliferating or differentiating cell conditions. To test the effect of gestational age on telomere length in the foetal brain, we ran a linear regression with relative telomere length as the outcome variable, sex as a covariate and gestational age (days) as the independent variable. Data normality was confirmed using the Shapiro–Wilk test. RNA-sequencing and downstream analyses were performed as described above.

### Figure generation

Figures were created using Prism7 (GraphPad, San Diego USA), the EnhancedVolcano package in R [67] and BioRender (Toronto, Canada).

## Results

### (i) Hippocampal progenitor cells demonstrate telomere shortening in response to serial passaging and the end-replication problem

**Fig. 2:**
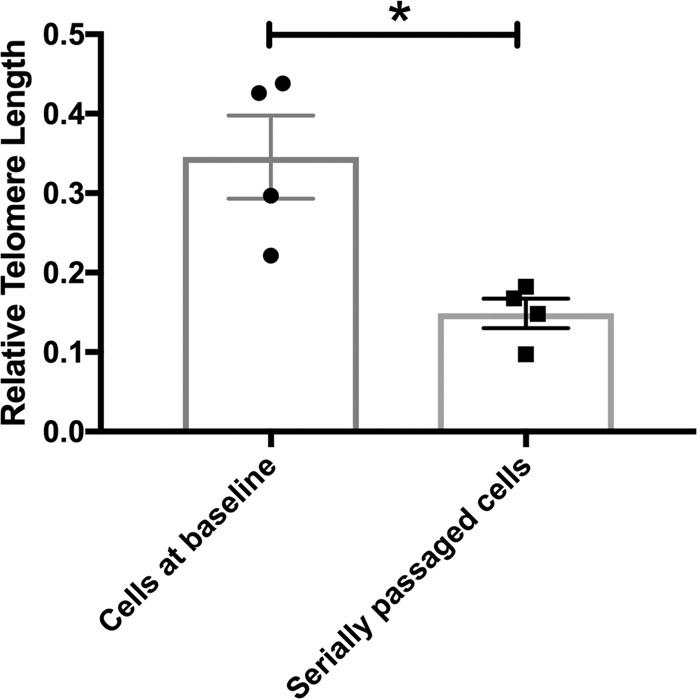
Telomeres are shorter in serially passaged hippocampal progenitor cells. This bar chart shows the relative telomere length in cells at baseline (P21) and serially passaged cells (P29). There is a significant reduction in telomere length in serially passaged cells relative to cells at baseline. Group differences were detected using an independent samples *t*-test. Significant differences were considered when *P* ≤ 0.05, indicated by *.

We compared relative telomere length in cells at baseline (P21) and in cells which had undergone serial passaging (P29). An independent samples *t*-test confirmed shorter telomere length in the serially passaged cells relative to cells at baseline (*t*(6) = 3.542, *P* = 0.012), Fig. 2.

To complement this finding, we confirmed that telomere shortening was likely being driven by the end-replication problem, as opposed to changes to the telomerase enzyme (another critical telomere regulator) during passaging. *hTERT* codes for the catalytic subunit of the telomerase enzyme and is tightly controlled, and closely associated with enzyme activity [68]. We performed a quantitative PCR to assess differences in the expression of telomerase reverse transcriptase (*hTERT*) in cells at baseline and serially passaged cells, and found consistently low levels of *hTERT* expression (relative to the reference gene, Vimentin), which did not differ between groups (*t*(6) = 1.151, *P* = 0.294); see Supplementary information, S10.

### (ii) Serially passaged hippocampal progenitor cells exhibit decreased cell proliferation

**Fig. 3:**
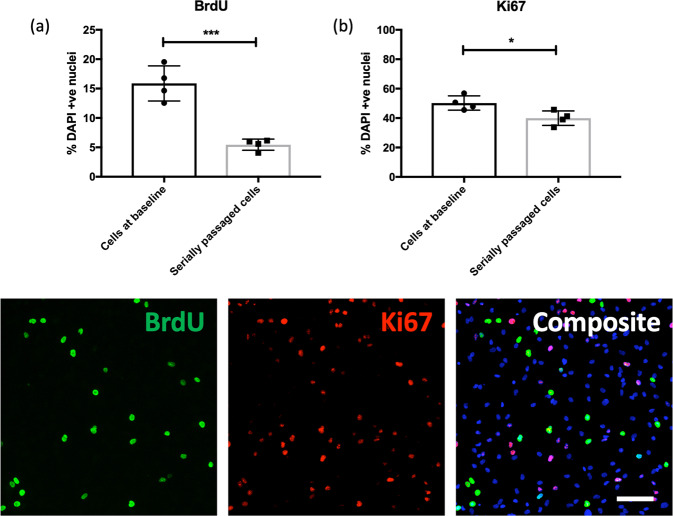
Proliferation rates are lower in serially passaged hippocampal progenitor cells. Bar charts (top) show the percentage of BrdU (**a**) and Ki67 (**b**) -positive cells relative to the percentage of DAPI stained nuclei (*y*-axis), in cells at baseline and serially passaged cells (*x*-axis). Each datapoint represents one biological replicate (*N* = 4). * represents an uncorrected *P* ≤ 0.05, and *** represents an uncorrected *P* ≤ 0.001. Each of the images (below) are representative of a field of immuno-stained cells, taken using a ×10 objective with the CellInsight High Content Screening Platform. Each composite image includes the nuclear marker DAPI in blue. Scale bar = 100  μm.

**Fig. 4:**
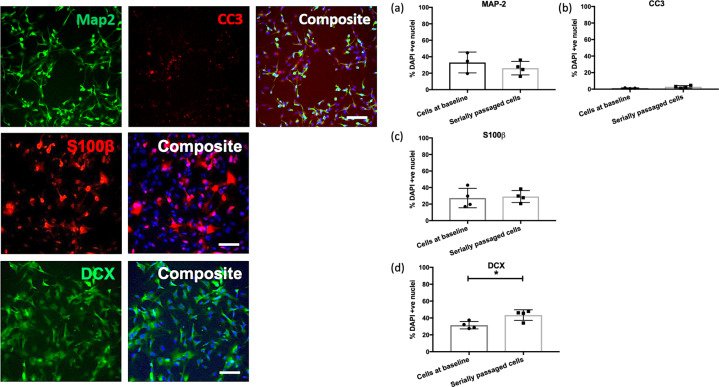
There are no differences in the rates of cell differentiation in serially passaged cells. The bar charts (right) show the percentage of MAP-2 (**a**), CC3 (**b**), S100β (**c**) and DCX (**d**) -positive cells relative to the percentage of DAPI stained nuclei (y-axis), in cells at baseline and serially passaged cells (x-axis). Each datapoint represents one biological replicate (*N* = 4). * represents an uncorrected *P* ≤ 0.05. Each of the images (left) are representative of a field of immuno-stained cells, taken using a ×10 objective with the CellInsight High Content Screening Platform. Each composite image includes the nuclear marker DAPI in blue. Scale bar = 100 μm.

Cells demonstrated a significant reduction in proliferation in association with telomere shortening, Fig. 3. Serially passaged cells exhibited significantly lower levels of proliferation relative to baseline cells, as marked using BrdU staining (two-sample *t*-test, *t*(6) = 6.663, *P* = 0.0006), a difference which remained significant after correcting for the number of cell markers tested (*P* ≤ 0.05). This difference was also supported by quantification of the proliferation marker Ki67 (two-sample *t*-test, *t*(6)  = 2.959, *P* = 0.025). We also observed a lower percentage of serially passaged cells stained with CC3 (indicative of lower rates of cell death), relative to cells at baseline (two-sample *t*-test, *t*(6) = 2.481, *P* = 0.047), however this effect did not survive multiple testing correction (*P* > 0.05); representative CC3 staining is shown in Fig. 4.

### (iii) Serially passaged hippocampal progenitor cells do not show differences in their rate of cell differentiation

We observed no differences (*P* > 0.05) in markers pertaining to glial cells (S100β), cell death (CC3) or more mature neurons (MAP-2), between cells at baseline and serially passaged cells. There was a small increase in the number of doublecortin-positive neurons observed in serially passaged cells (two-sample *t*(6) = 3.097, *P* = 0.021), though this difference did not survive multiple testing correction (*P* > 0.05), Fig. 4.

### (iv) Cell ageing is associated with vast transcriptional changes related to neurogenic processes, cellular senescence and inflammation

**Fig. 5:**
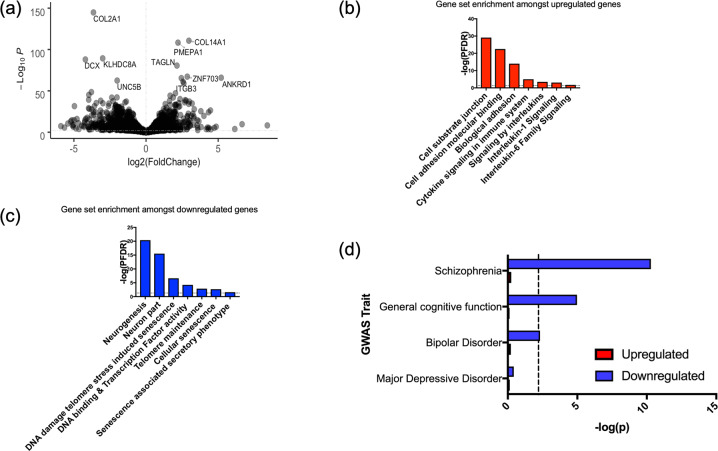
Differentially expressed genes in serially passaged cells relative to cells at baseline, and gene sets implicated in cell ageing. **a** A volcano plot summarising the RNA-sequencing results, where log2(Fold change) is shown on the x-axis, and the strength of the association given by −Log_10_(*P*), is shown on the *y*-axis. **b** Examples of gene sets which significantly overlap with the upregulated genes in our cell model. All gene sets represent those which surpassed a false discovery rate correction, *P*_FDR_ < 0.05, in our enrichment analysis, as marked by the dashed line. **c** Examples of gene sets which significantly overlap with the downregulated genes in our cell model. **d** A bar plot showing the genetic overlap between various traits as assayed by GWAS (*y*-axis) and genes either upregulated or downregulated in association with cell ageing. The strength of the association is shown on the *y*-axis (−log(*p*)). The dashed line represents the threshold of significance (corrected for the number of tests).

We identified 3281 transcripts which were differentially expressed in serially passaged cells relative to cells at baseline (*P*_FDR_ < 0.05), Fig. 5a. We found that 1687 genes were upregulated in serially passaged cells, and these genes were associated with cell adhesion. Enrichment amongst canonical pathways revealed an over-representation of genes related to inflammation and cytokine signalling, Fig. 5b. In agreement with previous work relating to the senescence associated secretory phenotype, amongst the upregulated transcripts we observed increased expression of the cytokine regulator, Nuclear Factor Kappa B (*NFKB1*) and increased interleukin-6 (*IL6*) levels (*P*_FDR_ < 0.05) [19]. See Supplementary Datasets for full results.

1594 genes were also downregulated in serially passaged cells. These genes were broadly associated with neurogenesis and nervous system development. Furthermore, enrichment amongst canonical pathways revealed an over-representation of genes related to telomeres, and cell senescence, Fig. 5c. Note that the downregulated gene expression changes were also enriched for genes affecting cell proliferation (GO_REGULATION_OF_CELL_POPULATION_PROLIFERATION, adj *P* = 0.00244125; see Supplementary dataset), which supports our cell staining data.

### (v) Genes downregulated in association with cell ageing overlap with those implicated in cognitive performance, schizophrenia and bipolar disorder risk

We found a significant overlap between genes which were downregulated in serially passaged cells (*P*_FDR_ < 0.05) and those implicated in: schizophrenia (*β* = 0.207, SE = 0.032, *P* = 5.105 × 10^−11^); bipolar disorder (*β* = 0.071, SE = 0.027, *P* = 0.005); and general cognitive function (*β* = 0.138, SE = 0.037, *P* = 1.057 × 10^−5^), based on GWAS results, Fig. 5d.

To ensure this result was not being inflated by the relatively large size of our gene set (*n* = 1594), or by an overrepresentation of genes related to tissue type, we performed a sensitivity analysis. From the transcripts surpassing our multiple testing threshold, we selected 500 which exhibited the highest fold change. In addition, when mapping SNPs to genes, we removed any genes which were not expressed in our cell line from the genomic annotation. When repeating gene-set enrichment analysis, we confirmed that downregulated transcripts remained significantly associated with each trait (*P* ≤ 0.01). We found no significant overlap between downregulated genes and those implicated in major depressive disorder, nor between upregulated genes and any of our traits.

### (vi) Telomere shortening may not occur during early neurodevelopment

Previous evidence suggests that telomere shortening and reduced rates of hippocampal neurogenesis are specific to the adult brain [42–44], but schizophrenia risk and cognitive function are also known to be affected by prenatal neurodevelopmental factors [69]. We performed a complementary experiment in order to clarify whether telomere shortening occurs in the human brain during early development in accordance with gestational age, and therefore, whether our cell model could also be recapitulating a neurodevelopmental cell process. To determine whether telomere shortening occurs in the developing brain, we assessed telomere length in foetal brain samples collected from various post-conception days (75–161 days), which is a measure that positively correlates with the number of cell divisions [70]. We did not find a significant correlation between telomere length and post-conception days (*β* = −0.003 [95% CI: −0.328 to 0.322], *P* = 0.985), nor an effect of sex (*β* = −0.001 [−0.011 to 0.009], *P* = 0.887), see Supplementary information, S11. This suggests that the effect we observe in our study, is unlikely to occur in early neurodevelopment, further supporting the specificity of this process to the postnatal brain.

## Discussion

Our study used serial passaging, an established method for modelling telomere shortening via the end replication problem, in human hippocampal progenitor cells. Serially passaged cells demonstrated reduced rates of cell proliferation, as determined by BrdU and Ki67 immunostaining. Telomere shortening was further accompanied by changes to 3281 transcripts, whereby downregulated genes overlapped with those implicated in cognition, schizophrenia and bipolar disorder.

The findings presented here support previous research performed in other cell model systems that reveal an intricate relationship between telomere length and cell replicative capacity related to the end-replication problem [17, 18, 57]. Our gene expression data further demonstrates an enrichment of transcript changes related to cell senescence, the senescence associated secretory phenotype, and interleukin-6 signalling. This is in agreement with previous research [19], and validates that our model is capturing meaningful biological changes related to telomere shortening and cell ageing. It also suggests that the decreased proliferation (marked by BrdU) and the nominally decreased cell death (marked by CC3; uncorrected *P* < 0.05) we observed in serially passaged progenitors, likely relates to early signs of cell senescence, i.e. a reduced number of new, healthy cells, and an accumulation of mitotically old, unhealthy cells that instead of apoptosing, demonstrate a proinflammatory phenotype.

In the context of neurogenesis, our findings extend prior work in animals and post-mortem brain which have shown a significant decline in markers of cell proliferation in the hippocampus in association with age [71, 72], by raising the possibility that telomere shortening is one potentially important age-related cellular mechanism. In contrast to some work [43, 51], we did not observe a robust effect of telomere shortening on rates of neuronal differentiation, as marked by doublecortin and MAP-2 in our cell staining data. However, amongst proliferating cells there was a strong downregulation of doublecortin at the transcript level in association with telomere shortening (Fig. 5a), which might suggest that there are very early, transient reductions in the rates of differentiation, which were not observable after the 7-day differentiation protocol used in our model.

Genetic and association studies have inferred a causal relationship between telomere length and cognition [31], but no study to-date, has demonstrated a cellular mechanism. Our work supports these findings and demonstrates that telomere shortening is associated with reduced hippocampal progenitor cell proliferation alongside changes to the expression of genes regulating general cognitive function. Our cell model and the gene-set enrichment results raise the possibility of a relationship between telomere shortening in human hippocampal progenitors and schizophrenia and bipolar disorder risk. Both schizophrenia and bipolar disorder share a common aetiology [73], and patients frequently exhibit smaller hippocampal volumes [25–27] and general cognitive deficits relating to attention, working memory, verbal learning and memory and executive functions [74]. In the context of our results, it is possible that telomere-induced reductions in hippocampal neurogenesis represent one mechanism that contributes to cognitive dysfunction amongst these patients. This notion is also supported by a study using mice, which demonstrates that a maternal infection results in offspring with schizophrenia-like behaviours, reduced rates of adult hippocampal neurogenesis, and neural progenitors with both shorter telomere length and reduced telomerase activity [75]. Interestingly, an environmental intervention (increased exercise) was able to normalise both abnormal cell phenotypes and behaviour in this model [75].

Our results add to the work of others (e.g. [75, 76]), and collectively, they raise the possibility that environmental factors with the potential to prevent premature telomere shortening may also have positive influences on hippocampal neurogenesis and cognitive function. Intriguingly, environmental interventions shown to reduce the rate of cognitive ageing [77], including exercise and energy restriction, have positive effects on both peripheral telomere length in humans [15, 78, 79] and hippocampal neurogenesis in animal models [80, 81]. Furthermore, diets rich in omega-3 fatty acids and antioxidants, have been associated with both increased rates of hippocampal neurogenesis and longer telomeres [32, 82, 83], as have some drugs, such as resveratrol and lithium [14, 84–86]. This warrants further study in order to determine whether environmental, dietary and pharmacological interventions could be useful in systematically targeting premature cell ageing and reduced hippocampal neurogenesis.

Despite the promising evidence shown here, there are a number of limitations to our study which should be acknowledged. First, we are measuring changes to telomere length and its effect on hippocampal neurogenesis using a cell model system acquired from a single donor. It is possible that individuals from different genetic backgrounds respond differently to the effects of telomere shortening [14], and as such, future work utilising induced pluripotent stem cell models from a range of donors would be beneficial in validating and extending our work. In addition, our in vitro model may lack construct validity due to the absence of mature cells and established intercellular networks, which are known to interact with progenitor cells and affect their development [87]. Second, although our cell model demonstrated telomere shortening and signs of cellular senescence, the effect was modest, with some serially passaged cells continuing to proliferate [10]. It is likely that a longer culture protocol will provide greater insights into the progressive and longer-term consequences of telomere shortening in hippocampal cells, and this is an important future consideration. Third, the cell model attempts to investigate the relationship between telomere shortening and adult hippocampal neurogenesis, in order to better understand age-related changes to the brain. However, alongside changes in telomere length, there may also be independent changes to the cell (e.g. the epigenetic landscape [88]), so future work will also be needed to further differentiate the causative effects of telomere shortening (e.g. via *TERT* knockout), from other age-related cell changes. Furthermore, our model makes use of foetal-derived neural progenitors, which although studies suggest do recapitulate the functions of adult neural stem cells, they may not do so entirely [89]. Moreover, our work in foetal brain indicates that despite the importance of early neurodevelopmental factors in the aetiology of schizophrenia [69], telomere shortening does not appear to occur during early brain development. Consequently, further work will be needed to confirm at what stages in postnatal life telomere shortening impacts on hippocampal neurogenesis and becomes important in the pathophysiology of psychiatric disease.

This work extends our understanding of the age-related molecular mechanisms influencing hippocampal neurogenesis, and provides support that telomere shortening may be an important process associated with psychiatric disorders and cognitive function. It also raises the possibility that there is a multisystemic relationship, in which telomere shortening contributes not only to the heightened rates of age-related disease amongst psychiatric disorder patients, but its putative effects on hippocampal neurogenesis suggest it could also represent a mechanism important in the pathophysiology of psychiatric disorders themselves. Future work should consider whether leukocyte telomere length could act as a useful peripheral biomarker to estimate changes in the rates of hippocampal neurogenesis in vivo, and whether environmental interventions could be used to simultaneously prevent premature telomere shortening and promote hippocampal progenitor proliferation, as we age.

## Funding and disclosure

Authors report no conflicts of interest. This work was funded by a Medical Research Council Skills Development Fellowship (MR/N014863/1) and a Psychiatry Research Trust Grant (grant reference: 92 Branthwaite) awarded to TRP, as well as by Medical Research Council funding (MR/N030087/1) awarded to ST. ABP is funded by a Rayne Foundation PhD studentship and by the National Institute for Health Research (NIHR) Mental Health Biomedical Research Centre, South London and Maudsley NHS Foundation Trust and King’s College London. The views expressed are those of the authors and not necessarily those of the NHS, the NIHR or the Department of Health and Social Care. The funding sources had no role in the study the design, in the collection, analysis and interpretation of data, in the writing of the report and in the decision to submit the article for publication. The human embryonic and foetal material was provided by the Joint MRC/Wellcome (MR/R006237/1) Human Developmental Biology Resource (www.hdbr.org).

## Supplementary information

Supplementary information

Supplementary Dataset
